# CerebrA, registration and manual label correction of Mindboggle-101 atlas for MNI-ICBM152 template

**DOI:** 10.1038/s41597-020-0557-9

**Published:** 2020-07-15

**Authors:** Ana L. Manera, Mahsa Dadar, Vladimir Fonov, D. Louis Collins

**Affiliations:** McConnell Brain Imaging Centre, Montreal Neurological Institute, McGill University, Montreal, H3A 2B4, 514-398-4227 Quebec (QC) Canada

**Keywords:** Diagnostic markers, Diagnostic markers, Brain imaging, Brain

## Abstract

Accurate anatomical atlases are recognized as important tools in brain-imaging research. They are widely used to estimate disease-specific changes and therefore, are of great relevance in extracting regional information on volumetric variations in clinical cohorts in comparison to healthy populations. The use of high spatial resolution magnetic resonance imaging and the improvement in data preprocessing methods have enabled the study of structural volume changes on a wide range of disorders, particularly in neurodegenerative diseases where different brain morphometry analyses are being broadly used in an effort to improve diagnostic biomarkers. In the present dataset, we introduce the Cerebrum Atlas (CerebrA) along with the MNI-ICBM2009c average template. MNI-ICBM2009c is the most recent version of the MNI-ICBM152 brain average, providing a higher level of anatomical details. Cerebra is based on an accurate non-linear registration of cortical and subcortical labelling from Mindboggle 101 to the symmetric MNI-ICBM2009c atlas, followed by manual editing.

## Abstract (summary)

**Table:** 

Measurement(s)	neuroantomical mapping • brain atlas • brain segmentation
Technology Type(s)	computational modeling technique
Sample Characteristic - Organism	Homo sapiens

Machine-accessible metadata file describing the reported data: 10.6084/m9.figshare.12472070

## Background & Summary

Brain atlases are widely recognized as important tools in research for the analysis of neuroimages. High spatial resolution magnetic resonance imaging (MRI) and improved data preprocessing have enabled the study of structural volume changes in a wide range of disorders. Anatomical atlases are central to the understanding of the brain anatomy and are the best resources to bring prior knowledge about anatomy into any computer vision methodology involved in various types of brain imaging analyses. Anatomical atlases can also be used to investigate potential disease-specific changes that occur in clinical cohorts compared with healthy populations, by providing information on region locations for various regions of interest. Analysis of fMRI data also routinely involves registration to a template and extraction of the average signal within various regions of interest within the corresponding anatomical atlases^1^.

The MNI-ICBM152 brain template^2^, from the Montreal Neurological Institute (MNI) is a crucial tool in neuroimage analysis. This multi-contrast atlas including T1w, T2w and PDw contrasts, was built recruiting brain scans from 152 young adults at 1.5 T. The 2009 edition uses group-wise non-linear registration for better alignment of cortical structures between subjects. The MNI-ICBM152 non-linear model has many advantages. It was created from a large number of subjects; hence it represents the average anatomy of the population and is not biased unlike single-subject models. In addition, the left-right symmetric version enables interpretation of asymmetries that might be found in an analysis.

Mindboggle-101 is the largest, publicly available set of manually labelled human brain images created from 101 human scans, labelled according to a surface-based cortical labelling protocol (DKT- Desikan-Killiany-Tourville labelling protocol)^3,4^. For the creation of the Mindboggle-101 dataset, developed to serve as brain atlas for use in labelling other brains, 101 T1-weighted (T1w) brain MRI images were selected and segmented based on a modification of the DKT cortical parcellation atlas^4^. These labels were then manually edited in agreement with the DKT protocol. Labelling was performed on the surface, yet, topographical landmarks visible in the folded surface were used to infer label boundaries. In addition, Mindboggle used non-cortical labels that were converted from Neuromorphometrics BrainCOLOR subcortex labels^4^.

The Cerebrum Atlas (CerebrA) includes co-registration of the Mindboggle atlas^3^ to the symmetric version of MNI-ICBM 2009c^2^ average template (at a resolution of 1 × 1 × 1 mm^3^) in addition to manual editing of cortical and subcortical labels. In the present dataset, we introduce an accurate non-linear registration of cortical and subcortical labelling from Mindboggle 101 to the symmetric MNI-ICBM2009c atlas followed by manual editing.

## Methods

### MNI-ICBM152 template

This section summarizes the details on generation of the nonlinear MNI-ICBM2009c average template. Further methodological details can be found in the original paper by Fonov *et al*.^2^. Within the ICBM project, MRI data from 152 young normal adults (18.5–43.5 years) were acquired on a Philips 1.5 T Gyroscan (Best, Netherlands) scanner at the Montreal Neurological Institute. The T1w data were acquired with a spoiled gradient echo sequence (sagittal acquisition, 140 contiguous 1 mm thick slices, TR = 18 ms, TE = 10 ms, flip angle 30°, rectangular FOV of 256 mm SI and 204 mm AP). The Ethics Committee of the Montreal Neurological Institute approved the study, and informed consent was obtained from all participants^2^.

**Fig. 1:**
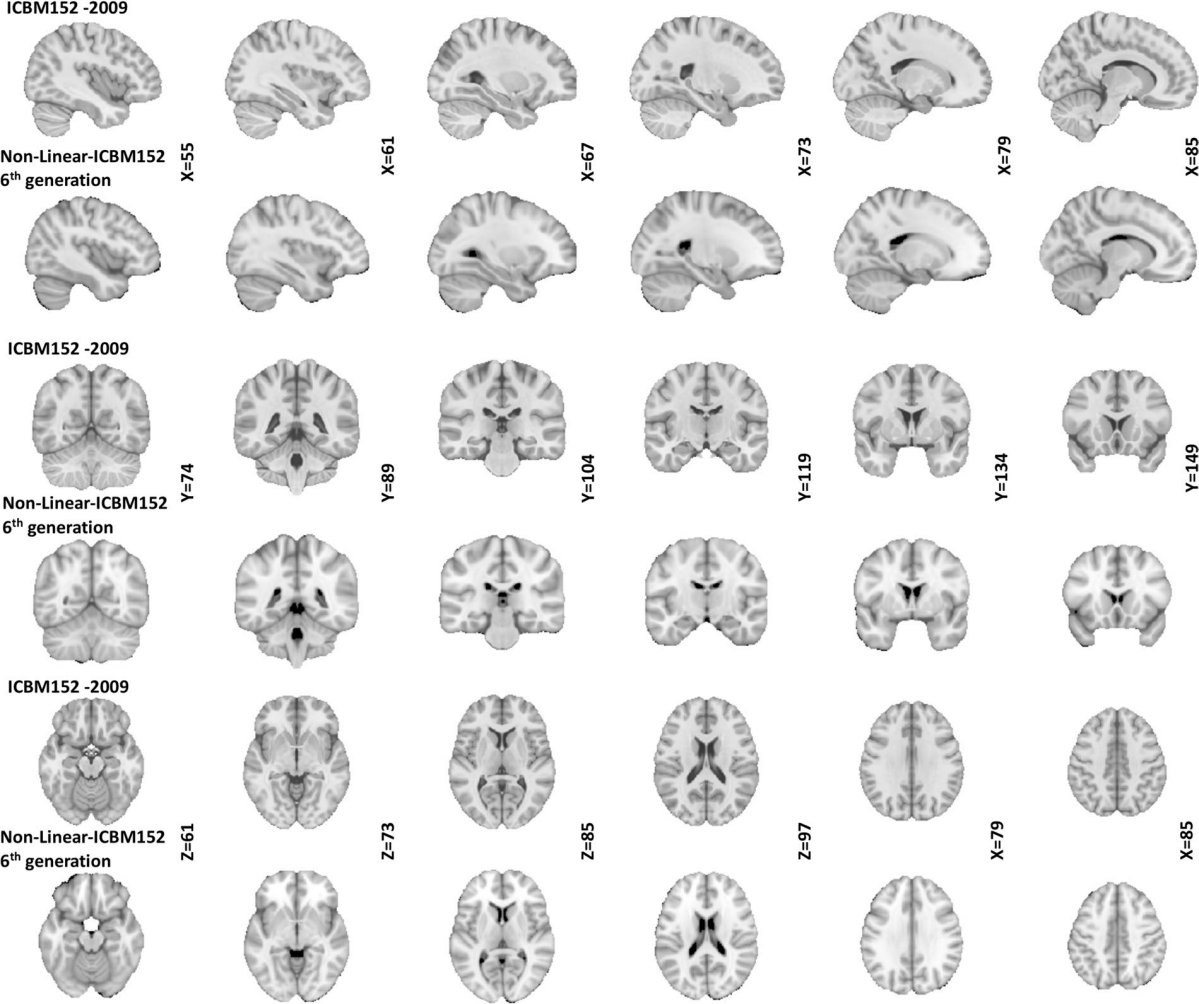
Comparison between the two versions of MNI-ICBM152 template. Rows 1, 3, and 5 show sagittal, coronal, and axial slices of the MNI-ICBM2009c template, respectively. Rows 2, 4, and 6 show sagittal, coronal, and axial slices of non-linear 6th generation template, respectively. Note the improved tissue contrast and cortical definition of the new template compared to the older 6^th^ generation version.

The following preprocessing steps were applied to all MRI scans prior to building the atlas: (1) N3 non-uniformity correction^5^; (2) linear normalization of each scan’s intensity to the range [0–100] by a single linear histogram scaling^6^; (3) automatic linear (nine parameters) registration to the ICBM 152 stereotaxic space^7^; and (4) brain mask creation^8^. Only the voxels within the brain volume after linear mapping into stereotaxic space were used for the nonlinear registration procedure described. The template described is generated through a hierarchical nonlinear registration procedure, with diminishing step sizes in each iteration until convergence and relies on the nonlinear registration using Automatic Nonlinear Image Matching and Anatomical Labelling (ANIMAL)^9^. The nonlinear versions of MNI-ICBM2009 (http://nist.mni.mcgill.ca/?p=904) have many advantages over widely used previous versions (i.e. MNI-ICBM non-linear 6^th^ generation; http://nist.mni.mcgill.ca/?p=858, https://fsl.fmrib.ox.ac.uk/fsl/fslwiki/Atlases). Besides, anatomical variability still remains after linear transformation to stereotaxic space, therefore sulci and gyri remain blurred in previous versions^10^ (Fig. 1).

### Mindboggle-101

This section summarizes the generation of the original Mindboggle-101 atlas and additional methodological details can be found in the original paper by Klein and Tourville 2012^3^. The authors started with publicly accessible T1-w MRI scans selected from 101 healthy participants. Scanner acquisition and demographic information can be found in Klein 2012^3^ and are also available on the http://mindboggle.info/data website. The data sets that comprise the Mindboggle-101 include the 20 test–retest subjects from the “Open Access Series of Imaging Studies” data^11^, the 21 test–retest subjects from the “Multi-Modal ReproducibilityResource”^12^, with two additional subjects run under the same protocol in 3 T and 7 T scanners, 20 subjects from the “Nathan Kline Institute Test–Retest” set, 22 subjects from the “Nathan Kline Institute/Rockland Sample”, the 12 “Human Language Network” subjects^13^, the Colin Holmes 27 template^14^, two identical twins, and one brain imaging colleague.

T1-w MRI volumes were preprocessed and segmented and then, cortical surfaces were generated using FreeSurfer’s standard recon-all image processing pipeline^15,16^. FreeSurfer then automatically labelled the cortical surface using its DKT cortical parcellation atlas^4,17^. Vertices along the cortical surface are assigned a given label based on local surface curvature and average convexity, prior label probabilities, and neighbouring vertex labels. FreeSurfer automatically labelled the cortical surface using its DKT cortical parcellation atlas for 54 of the brains in the Mindboggle-101 data set. The region definitions of the labelling protocol represented by the DKT atlas are described by Desikan *et al*.^4^. These labels were then manually edited in agreement with the DKT protocol with 31 cortical regions per hemisphere as described by Klein and Tourville^3^. Then, the first 40 brains that labelled were selected to train a new FreeSurfer cortical parcellation atlas representing the DKT protocol (see http://surfer.nmr.mgh.harvard.edu/fswiki/FsTutorial/GcaFormat; S’egonne *et al*.^17^; Desikan *et al*.^4^ for details regarding the algorithm that generates the atlas and how it is implemented). The resulting atlas was named “DKT40 classifier atlas” which then automatically generated the initial set of cortical labels for the remaining 47 brains in the data. Finally, Mindboggle data includes non-cortical labels that were converted from the Neuromorphometrics BrainCOLOR subcortex labels (i.e., http://Neuromorphometrics.com/). Details on the original labels included in Mindboggle-101 can be found in https://mindboggle.readthedocs.io/en/latest/labels.html. *OASIS-30_Atropos_template* and *OASIS-TRT-20_jointfusion_DKT31_CMA_labels_in_OASIS-30_v2* were the template and atlas files that were used for registration to the MNI-ICBM2009 template and manual correction.

### Atlas registration and manual label editing

The Mindboggle-101 average template was first linearly and then non-linearly registered to the symmetric version of MNI-ICBM (MNI-ICBM2009c) template. In both registrations, the Mindboggle-101 template was used as the source image, and the MNI-ICBM2009c template was used as the target image. The linear registration was performed with 9 parameters (-lsq9, 3 for translation, 3 for rotation, and 3 for scaling), using *bestlinreg_s2* pipeline from the MINCTools^18^. The resulting image was then nonlinearly registered to MNI-ICBM2009c template using the ANTs diffeomorphic registration pipeline^19^, providing both source and target masks. Using the obtained nonlinear transformation, the Mindboggle-101 atlas labels were also resampled and registered to MNI-ICBM2009c template, using the label resampling option from itk_resample tool (i.e.,–label, applying a nearest neighbor interpolator for discrete labels). The quality of the registration was visually assessed by overlaying the registered Mindboggle-101 and MNI-ICBM2009c templates as well as the registered Mindboggle-101 atlas to ensure accurate transformation of the labels to the MNI-ICBM2009 template. Any remaining inaccuracies were manually corrected on the right hemisphere using the interactive software package Display, part of the MINC Tool Kit, developed at the McConnell Brain Imaging Center of the Montreal Neurological Institute (https://www.mcgill.ca/bic/software/visualization/display, https://github.com/BIC-MNI). The program allows simultaneous viewing and segmentation in the coronal, sagittal and axial planes, as well as intensity thresholding, label filling, dilation, and erosion. The corrections were performed by A.M., a neurologist with 10 years of experience in reading and assessment of MRIs. Prior to performing these corrections, A.M. received additional training on performing manual segmentations using Display by an anatomist expert both in using Display and MRI segmentation. These corrections mainly involved improvements of boundaries between neighboring regions, addition of missing voxels in some structures (detailed in Table 1) and improving the continuity of voxels within each region. Afterwards, labels were flipped onto the left hemisphere and then visual inspection on each structure was performed. In detail, thickness and boundaries of all 51 cortical and subcortical labels from each hemisphere were improved using intensity thresholds with manual painting using MNI Display. Details on the significant edits that were made for particular structures are provided in Table 1.

**Table 1:** Original label numbers from Mindboggle with new label numbers.

MB ID	Label Name	CerebrA ID		Notes	Kappa
		RH Labels	LH Labels		
2002	Caudal Anterior Cingulate	30	81		0.79
2003	Caudal Middle Frontal	42	93	Improved distinction from Precentral	0.73
2005	Cuneus	43	94		0.67
2006	Entorhinal	36	87	Improved delimitation	0.78
2007	Fusiform	24	75		0.77
2008	Inferior Parietal	10	61		0.75
2009	Inferior temporal	3	54	Removed dorsal part MT	0.72
2010	Isthmus Cingulate	33	84		0.79
2011	Lateral Occipital	34	85		0.76
2012	Lateral Orbitofrontal	7	58		0.8
2013	Lingual	12	63		0.75
2014	Medial Orbitofrontal	15	66		0.72
2015	Middle Temporal	28	79	Added dorsal part	0.72
2016	Para hippocampal	18	69		0.86
2017	Paracentral	16	67		0.77
2018	Pars Opercularis	32	83		0.77
2019	Pars Orbitalis	44	95		0.8
2020	Pars Triangularis	22	73		0.76
2021	Pericalcarine	6	57		0.6
2022	Postcentral	13	64		0.82
2023	Posterior Cingulate	47	98		0.8
2024	Precentral	35	86		0.84
2025	Precuneus	31	82		0.8
2026	Rostral Anterior Cingulate	8	59		0.72
2027	Rostral Middle Frontal	1	52	Improved delimitation from CMF	0.74
2028	Superior Frontal	38	89		0.82
2029	Superior Parietal	9	60	Improved delimitation from Precuneus and IP	0.72
2030	Superior Temporal	45	96	Added dorsal part limiting with IP and Supramarginal	0.87
2031	Supramarginal	51	102		0.81
2034	Transverse Temporal	14	65		0.85
2035	Insula	23	74		0.88
16	Brainstem	11	62	Completed filling, removed labelled voxels out of actual brainstem and removed CWM labels in brainstem area.	0.65
14	Third Ventricle	29	80		0.68
15	Fourth Ventricle	37	88	Missing label. Manually delimited using CSF threshold.	0.39
85	Optic Chiasm	17	68	Almost inexistent label and out of place in original labelling Completed OC and tracts (originally labelled as Ventral Diencephalon)	0
43	Lateral Ventricle	41	92	Improved continuity of labelled voxels	0.89
44	Inferior Lateral Ventricle	5	56		0.12
45	Cerebellum Gray Matter	46	97	Completed filling using threshold for CGM, removed cerebellum labels out of area (within brainstem and vermis area)	0.83
46	Cerebellum White Matter	39	90	Improved according threshold for CWM, removed labels in brainstem and vermis.	0.73
49	Thalamus	40	91		0.97
50	Caudate	49	100	Completed filling using threshold	0.84
**51**	**Putamen**	**21**	**72**	**Corrected uniformity using threshold**	**0.87**
52	Pallidum	27	78	Improved delimitation between putamen and pallidum	0.83
**53**	**Hippocampus**	**48**	**99**		**0.69**
54	Amygdala	19	70		0.64
**58**	**Accumbens Area**	**4**	**55**		**0.76**
60	Ventral Diencephalon	26	77		0.93
**92**	**Basal Forebrain**	**25**	**76**		**0.82**
630	Vermal lobules I-V	50	101	Improved delimitation with other vermal lobules and cerebellar hemispheres	0.66
**631**	**Vermal lobules VI-VII**	**2**	**53**	**Improved delimitation with other vermal lobules and cerebellar hemispheres**	**0.38**
632	Vermal lobules VIII-X	20	71	Improved delimitation with other vermal lobules and cerebellar hemispheres	0.44

Table is showing specific corrections that were made to some structures for CerebrA and the agreement between the two labelling methods (Dice Kappa coefficient).

Abbreviations: MB: Mindboggle-10; Vol: volume; MT: middle temporal; CMF: caudal middle frontal; IP: inferior parietal; CWM: cerebellar white matter; CSF: cerebrospinal fluid; OC: optic chiasm; CGM: cerebellar grey matter.

## Data Records

CerebrA probabilistic atlas, including the corresponding T1w template, as well as segmentations of labels are available at G-Node (10.12751/g-node.be5e62)^20^, TemplateFlow (https://github.com/templateflow/tpl-MNI152NLin2009cSym) and on http://nist.mni.mcgill.ca/?p=904. All imaging data are in compressed MINC^21,22^ and NIfTI formats. The registration and resampling scripts, the obtained transformations and the final Mindboggle-101 atlas labels registered to the MNI-ICBM2009 template are also available at 10.12751/g-node.be5e62. We invite contributions by other researchers, in terms of alternative opinions on labeling of included structures.

The template can be downloaded from TemplateFlow either with datalad:

$ datalad install -r ///templateflow $ cd templateflow/tpl-MNI152NLin2009cSym/ $ datalad get -r *

or python:

$ pip install templateflow

from templateflow import api api.get(‘MNI152NLin2009cSym’)

## Technical Validation

### Comparison between atlases

Dice Kappa similarity index was used to assess the degree of agreement between the CerebrA labels and the original Mindboggle-101 labels, after registration to the MNI-ICBM2009 template. The label agreement before and after manual correction is included to demonstrate the improvement achieved by manual correction. Dice Kappa measures the proportion of the number of voxels that are common between the two masks, over the total number of voxels within the masks, and is defined as:

$${\rm{\kappa }}=2\frac{{V}_{1}\cap {V}_{2}}{{V}_{1}+{V}_{2}}$$

where κ denotes the Dice Kappa coefficient, and *V*_1_ and *V*_2_ denote the two volumes under comparison. A Dice Kappa of 1 implies perfect agreement, whereas a Dice Kappa of 0 implies no overlap between the two masks.

**Fig. 2:**
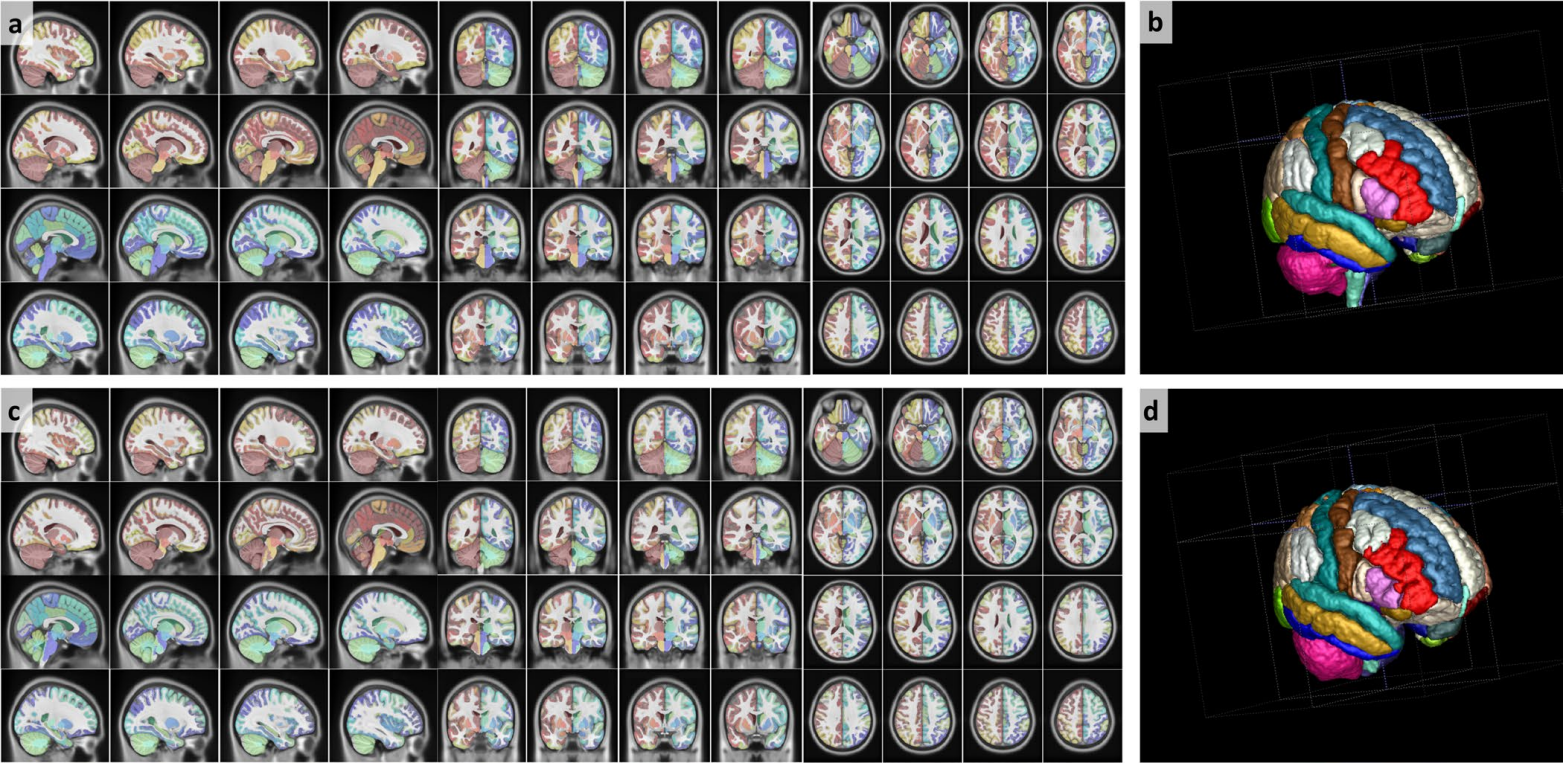
Warped CerebrA atlas (**a**,**b**) and Mindboggle-101 atlas (**c**,**d**) overlaid on the ICBM152 non-linear 2009 symmetric average MRI template. Note the improved label alignment on the cortical structures.

**Fig. 3:**
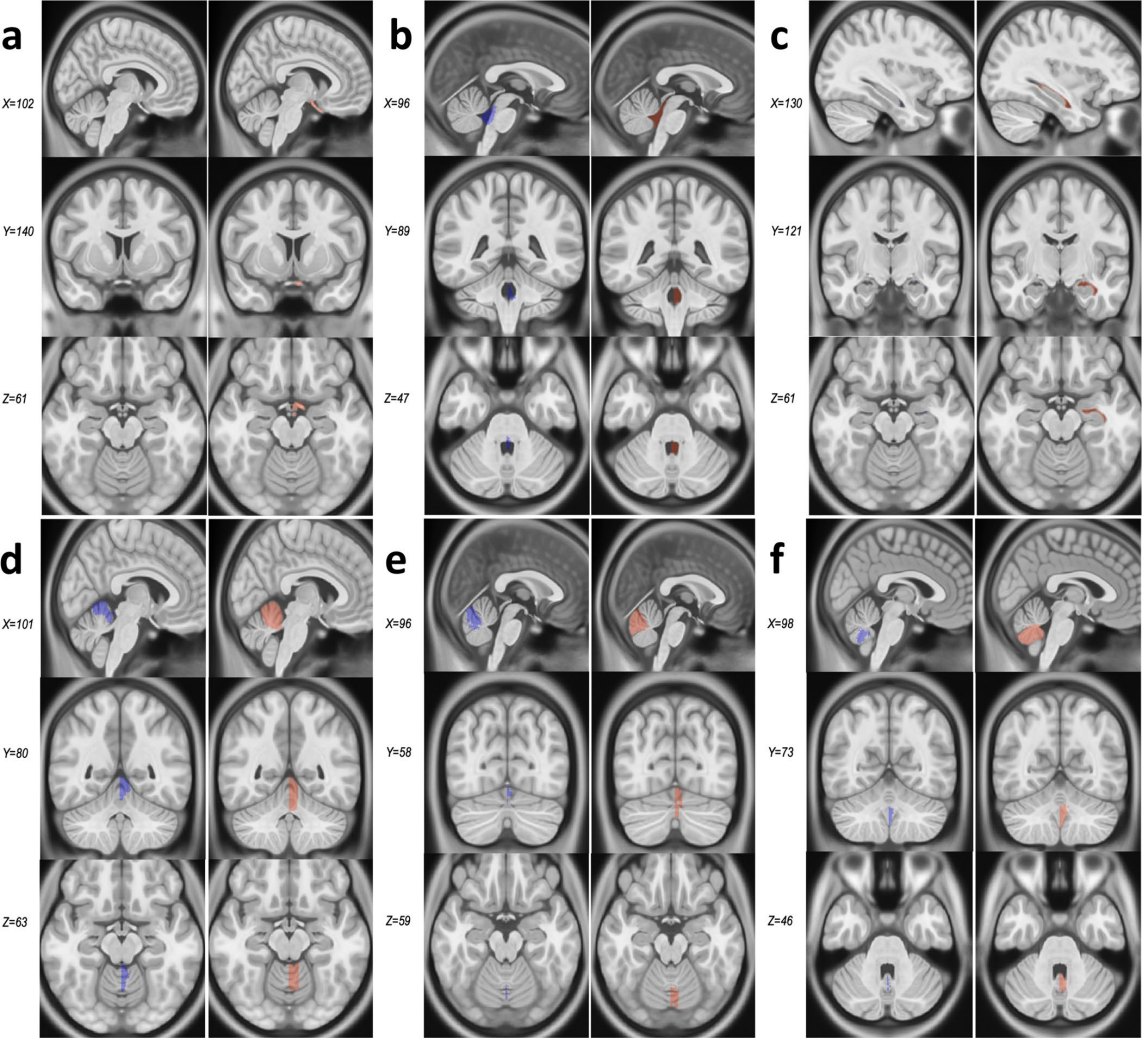
Comparison between Mindboggle-101 and CerebrA for structures with Dice Kappa < 0.6. Panel a. Optic chiasm. Panel b. Fourth ventricle. Panel c. Inferior lateral ventricle. Panels d–f. Cerebellar vermis lobules. For each structure, the column on the left (blue) represents the original labels from Mindboggle-101, warped onto the ICBM152 symmetric template, and the right column (pink) represents CerebrA’s right sided corresponding labels, on the same template.

When comparing CerebrA to original labels from Mindboggle-101 (Fig. 2) registered to ICBM152, the average Dice Kappa value was κ = 0.73 ± 0.18 (Table 1). The structures with relatively lower Dice Kappa (κ < 0.6) corresponded to the structures that needed the most correction such as the optic chiasm, inferior lateral ventricles, fourth ventricle and cerebellar vermis. The optic chiasm label was barely found in the original Mindboggle-101 registered to ICBM152 and most of it was misaligned with regards to the actual structure. To ensure that this inaccuracy was not caused by the nonlinear registration process, we further inspected the original Mindboggle-101 template and label atlas and found similar issues. For CerebrA, the optic chiasm label was redefined trying to achieve continuity amongst optic chiasma itself and optic tracts (Fig. 3, panel a). Then, the inferior lateral ventricles and fourth ventricle boundaries were improved using a threshold to differentiate CSF from parenchyma (Fig. 3, panels b and c). And finally, cerebellar vermis labels were redefined for right and left side (Fig. 3, panels d–f).

**Fig. 4:**
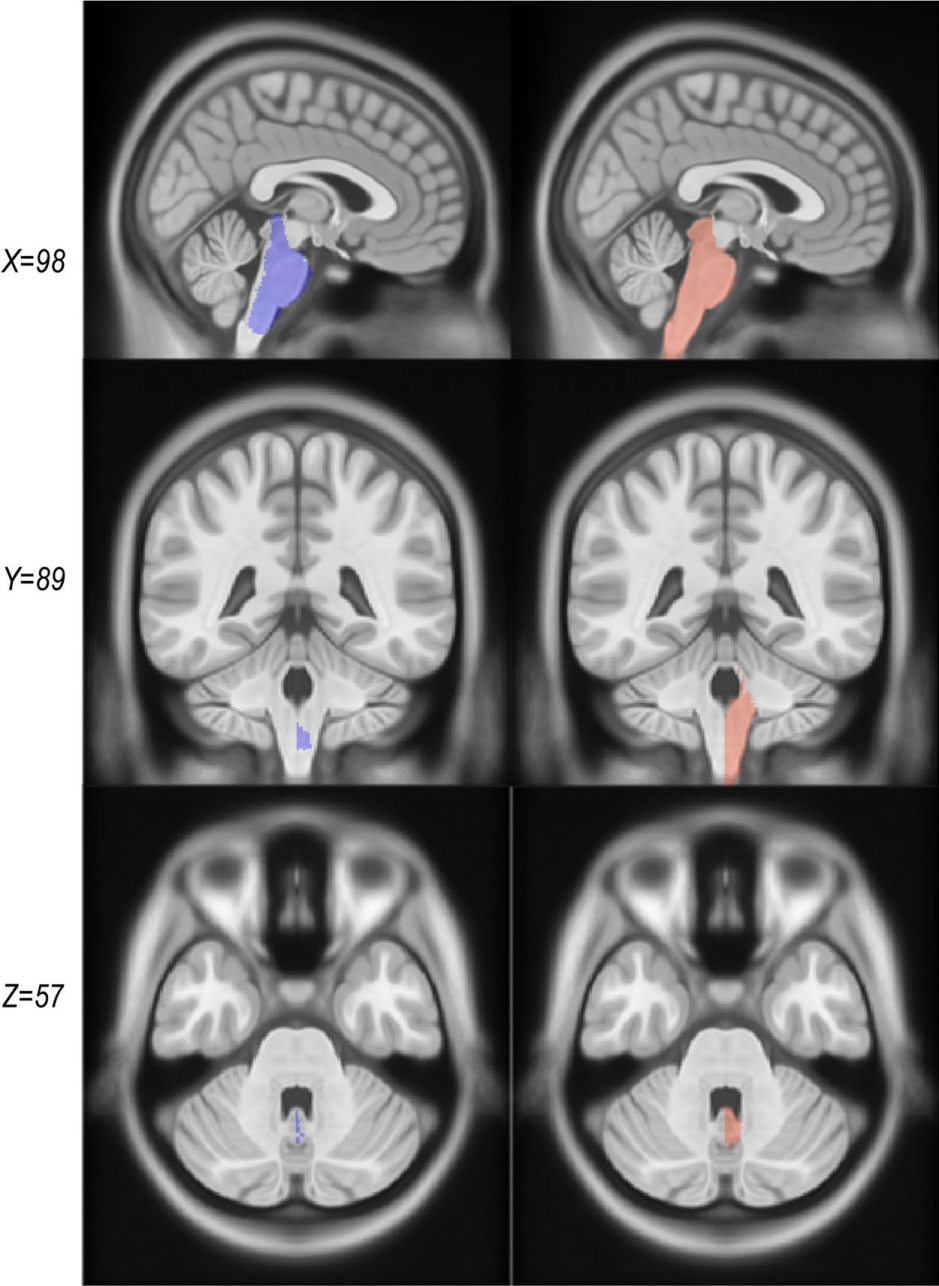
Comparison between warped Mindboggle-101 labels and CerebrA labels for the brainstem. The column on the left (purple) represents the original labels from Mindboggle-101 and the right column (coral) represents CerebrA’s right sided corresponding labels.

Another significant change in CerebrA from the original warped labels was the brainstem label definition. The brainstem area was manually redefined for the right side and then flipped in the same procedure as all the labels considering the symmetrical feature of the ICBM152 2009c^2^ template. In addition, boundaries between brainstem and fourth ventricle were carefully defined using the CSF intensity threshold, cerebellar white matter labels within the brainstem area were removed and rostral brainstem delimitation was improved (Fig. 4).

### Inter-rater and intra-rater variability assessments

To assess intra-rater variability, the rater repeated the process of manual correction for 10 randomly selected regions, and the results were compared against the previously corrected masks. The mean Dice-Kappa values between the two masks were 0.88 ± 0.03. To assess inter-rater variability, the same 10 selected regions were rated a second time by another independent rater and these results were also compared against the previously corrected masks, yielding a mean inter-rater Dice-Kappa of 0.83 ± 0.05.

### Volumes of cortical and subcortical structures

**Fig. 5:**
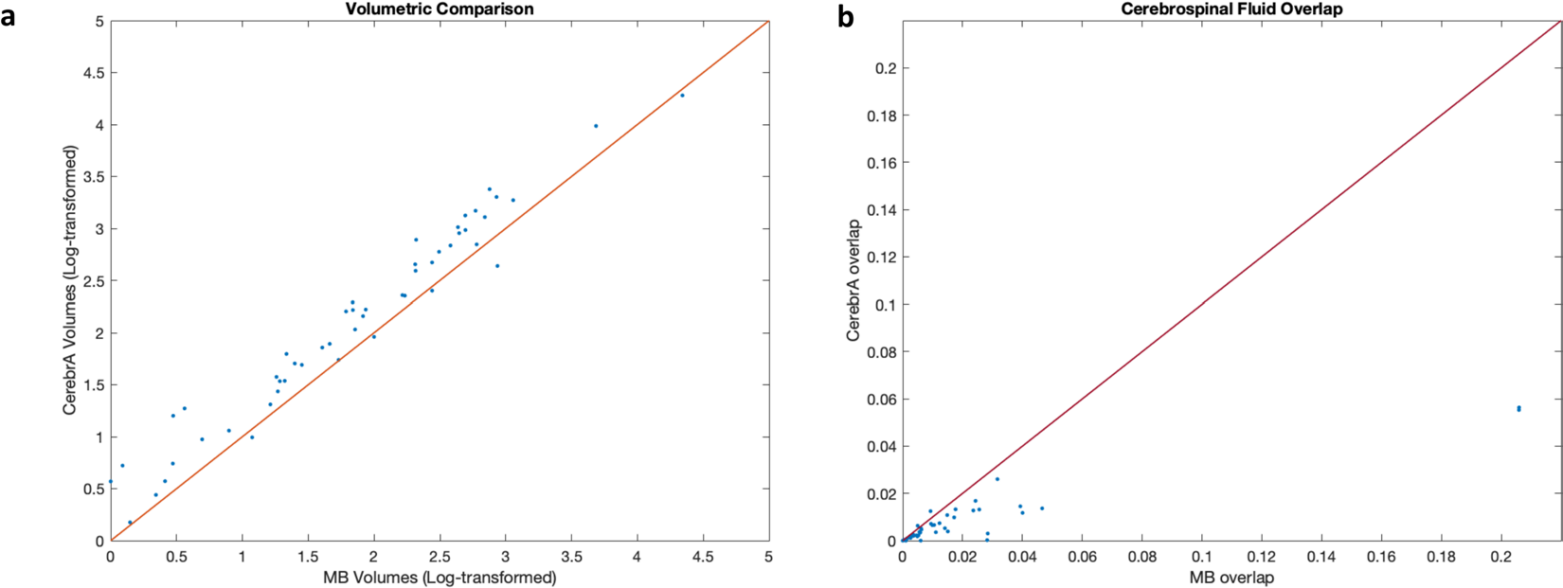
(**a**) Correlation plot between CerebrA and Mindboggle-101 volumes. For better visualization and to achieve a normal distribution, the volumes have been log-transformed. Correlation coefficient R = 0.9657, P value < 0.001. (**b**) Plot showing the proportion of overlap (the number of voxels in the specific mask overlapping with the CSF mask/the total number of voxels inside the specific mask) between the atlas labels and CSF for both atlases. MB: Mindboggle-101. CSF: CerebroSpinal Fluid.

Region volumes were calculated for all cortical and subcortical structures before and after performing the manual correction by summing up the number of voxels within each label (in CCs). These volumes were then log-transformed to achieve normal distribution to enable comparisons between the two sets of volumes. Figure 5a shows the correlation plot between the log-transformed CerebrA and Mindboggle volumes. Although the volumes were strongly and significantly correlated (R = 0.9657, P value < 0.001), overall volumes estimated with CerebrA were larger than those estimated with Mindboggle-101 (Fig. 5a). The volumes estimated per structure using Mindboggle and CerebrA segmentation are listed in Table 2.

**Table 2:** Volume per structure using Mindboggle-101 and CerebrA segmentations.

Label Name	Volume (cc)		Label Name	Volume (cc)	
	MB	CerebrA		MB	CerebrA
Caudal Anterior Cingulate	2.75	3.65	Superior Parietal	12.97	19.33
Caudal Middle Frontal	9.1	13.22	Superior Temporal	20.25	25.35
Cuneus	4.97	8.07	Supramarginal	12.2	16.03
Entorhinal	2.56	3.21	Transverse Temporal	1.45	1.88
Fusiform	10.48	13.47	Insula	8.33	9.52
Inferior Parietal	17.73	26.19	Brainstem	9.17	17.01
Inferior temporal	15.1	16.22	Third Ventricle	0.6	1.1
Isthmus Cingulate	3.27	4.43	Fourth Ventricle	0.51	0.77
Lateral Occipital	14.97	22.81	Optic Chiasm	0	0.77
Lateral Orbitofrontal	11.1	15.04	Lateral Ventricle	8.16	9.57
Lingual	9.13	12.36	Inferior Lateral Ventricle	0.09	1.06
Medial Orbitofrontal	5.28	8.88	Cerebellum Gray Matter	75.85	71.23
Middle Temporal	16.77	28.29	Cerebellum White Matter	17.86	13
Para hippocampal	2.36	2.71	Thalamus	10.48	10.04
Paracentral	5.94	8.24	Caudate	4.28	5.64
Pars Opercularis	5.79	7.67	Putamen	5.4	6.63
Pars Orbitalis	2.61	3.64	Pallidum	1.93	1.7
Pars Triangularis	5.29	8.2	Hippocampus	4.64	4.69
Pericalcarine	2.8	5.03	Amygdala	1	1.65
Postcentral	13.1	18.19	Accumbens Area	0.41	0.55
Posterior Cingulate	3.99	5.41	Ventral Diencephalon	6.39	6.1
Precentral	16.13	21.39	Basal Forebrain	0.16	0.19
Precuneus	13.79	18.77	Vermal lobules I-V	3.05	4.5
Rostral Anterior Cingulate	2.52	3.83	Vermal lobules VI-VII	0.61	2.32
Rostral Middle Frontal	13.77	21.74	Vermal lobules VIII-X	0.75	2.57
Superior Frontal	38.87	52.82			

Abbreviations: MB: Midboggle-101.

### Overlap with CSF

Using the CSF mask of the MNI-ICBM152 template, the number of voxels within each label that overlapped with the CSF was calculated to assess which template had a lower overlap with CSF. The four ventricular regions (i.e. lateral ventricles, inferior lateral ventricles, 3^rd^ and 4^th^ ventricles) were excluded from this analysis. Figure 5b shows the proportion of overlap of each of the labels with the CSF for each atlas; i.e. the number of voxels in the specific mask overlapping with the CSF mask divided by the total number of voxels inside the specific mask. Overall, Mindboggle-101 showed greater overlap of cortical and subcortical structures with CSF (Fig. 5b). Cerebellar vermal lobule regions (630 and 631) from Mindboggle-101 had the highest degree of overlap (20%) with the CSF, due to a combination of misalignment and over-segmentation errors (see Fig. 2c).

## Usage Notes

Atlases are sometimes used to compare individual subjects. Such comparisons, made based on average templates and corresponding atlases, are by nature prone to errors caused by registration of a subject’s brain to the template. Such errors are dependent on the individual scans, and are generally greater in presence of pathologies such as tumors, lesions, severe atrophy, etc.^18^. Therefore, these types of errors might introduce systematic biases in the findings, and great care should be taken to assess registration accuracy when performing such analyses.

## Data Availability

The scripts used to perform both the linear and nonlinear registrations (including the ANTs code with all the selected registration parameters), the obtained transformations that were used to register the DKT atlas to the MNI-ICBM2009c template, the code for resampling the labels based on these transformations, as well as the registered DKT atlas in the MNI space, after applying the transformations are available at https://gin.g-node.org/anamanera/CerebrA/src/master/.
